# Perioperative dexmedetomidine and renal outcomes in adult cardiac surgery: an updated systematic review and meta-analysis

**DOI:** 10.3389/fmed.2025.1737121

**Published:** 2026-01-16

**Authors:** Jie Wen, Fenglin Jiang

**Affiliations:** 1 Department of Emergency, Clinical Medical College & Affiliated Hospital of Chengdu University, Chengdu, Sichuan, China; 2 Department of Nursing, Pengzhou Peoples’s Hospital, Pengzhou, Sichuan, China

**Keywords:** acute kidney injury, cardiac surgery, dexmedetomidine, meta-analysis, renal outcomes

## Abstract

**Background:**

Acute kidney injury (AKI) is a common complication following cardiac surgery, associated with increased morbidity and mortality. Dexmedetomidine (DEX), a highly selective α-2 adrenoceptor agonist, has shown potential renal protective effects, but evidence remains inconsistent. This study aims to evaluate the efficacy of DEX in preventing AKI and improving renal outcomes in cardiac surgery patients through a systematic review and meta-analysis of randomized controlled trials (RCTs).

**Methods:**

A comprehensive search of PubMed, Cochrane Library, Embase, and Web of Science was conducted until April 2025. PICOS criteria were applied to select studies comparing DEX with placebo/normal saline in cardiac surgery patients. Primary outcomes included AKI incidence; secondary outcomes encompassed intraoperative parameters, postoperative recovery, and complications.

**Results:**

Bibliometric analysis highlighted China and the USA as leading contributors, with emerging trends in pediatric and mechanistic research. Among 16 RCTs (*n* = 2,882), DEX significantly reduced AKI incidence [RR 0.58; 95% CI 0.37 to 0.91; *I*^2^ = 74%, *p* = 0.02], particularly at 0.6–0.1 μg/kg/h doses [RR 0.43; 95% CI 0.26 to 0.71; *I*^2^ = 0%, *p* = 0.001]. Subgroup analysis revealed 0.4 μg/kg/h doses failed to yield a statistically significant benefit [RR 0.65; 95% CI 0.36 to 1.17; *I*^2^ = 84%; *p* = 0.15]. DEX also shortened ICU stay [MD −1.23; 95% CI −2.17 to −0.30; *I*^2^ = 93%; *p* = 0.01], mechanical ventilation duration [MD −1.24; 95% CI −2.15 to −0.33; *I*^2^ = 97%; *p* = 0.008], and hospital stays [MD −0.33; 95% CI −0.54 to −0.13; *I*^2^ = 86%; *p* = 0.01]. However, it did not affect mortality or intraoperative times.

**Conclusion:**

DEX demonstrates significant renal protection and improves postoperative recovery in cardiac surgery patients, though optimal dosing requires further investigation. These findings support its integration into perioperative protocols but underscore the need for standardized dosing regimens.

**Systematic review registration:**

Identifier, INPLASY2025120019.

## Graphical abstract

**Figure:**
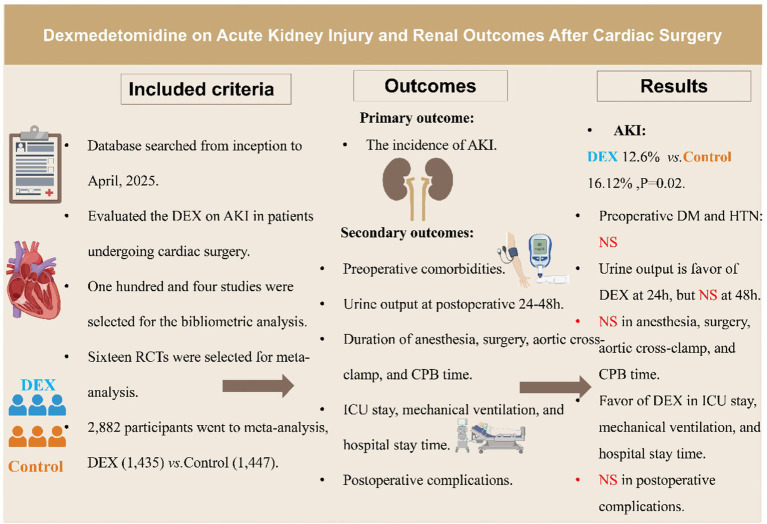
DEX, dexmedetomidine; NS, not significant; CPB, cardiopulmonary bypass; ICU, intensive care unit; DM, diabetes mellitus; HTN: hypertension.

## 1 Introduction

Cardiac surgery-associated AKI (CSA-AKI) occurs in 20–30% of patients, significantly elevating risks of chronic kidney disease and mortality (1–3). The pathophysiology involves ischemia–reperfusion injury, inflammation, and oxidative stress, exacerbated by cardiopulmonary bypass (CPB) (4, 5). The development of consensus criteria for AKI definition has enhanced our awareness of this possibly underdiagnosed complication after cardiac surgery. These criteria include Risk-Injury Failure-Loss-End-stage renal disease (RIFLE), acute kidney injury network (AKIN), and kidney disease improving global outcomes (KDIGO), which are used to define AKI based on increase in serum creatinine and/or reduced urinary output (6–9). Despite advances in surgical techniques, effective pharmacologic interventions remain limited.

Dexmedetomidine (DEX), a highly selective α-2 adrenoceptor agonist, has emerged as a promising adjunct due to its anti-inflammatory, anti-apoptotic, and hemodynamic-stabilizing properties (10, 11). Preclinical study demonstrate that DEX attenuates renal I/R injury, by up-regulating Sirtuin 3 (SIRT3) to inhibit mitochondrial damage and cell apoptosis (12). Clinically, however, evidence is conflicting. While study reduced AKI incidence with DEX (13), others show no benefit (14), potentially due to variability in dosing and patient populations (15). Though, previous study investigated the renal protection outcomes of DEX in cardiac surgery, the comprehensive analysis is lacking.

This study integrates bibliometric analysis and meta-analysis to address three key gaps in current literature. First, previous reviews have overlooked subgroup analyses stratified by DEX dosage (e.g., 0.2–1.0 μg/kg/h), a limitation that may account for the inconsistent outcomes reported across studies; second, few studies correlate DEX’s renoprotective effects with biomarkers (e.g., neutrophil gelatinase-associated lipocalin, NGAL, and cystatin C); third, bibliometric analysis identifying geographic disparities in research contributions, mapping evolving research themes, and contextualizing the meta-analysis findings within the broader academic landscape. Based on these identified gaps, we hypothesize that perioperative administration of DEX reduces the incidence of AKI and improves secondary clinical outcomes, with these effects exhibiting a dose-dependent pattern.

## 2 Methods

This study was conducted in accordance with the PRISMA (Preferred Reporting Items for Systematic Reviews and Meta-Analyses) and AMSTAR (Assessing the Methodological Quality of Systematic Reviews) guidelines (16, 17). The meta-analysis was prospectively registered in the INPLASY database (INPLASY2025120019).

### 2.1 Search strategy

A comprehensive literature search was performed across multiple electronic databases, including PubMed, Cochrane Library, Embase, and Web of Science, from inception until April 2025. The search strategy was designed using the PICOS (Patient, Intervention, Comparison, Outcome, Study design) framework, incorporating the following Boolean search terms: “Dexmedetomidine,” AND “Acute kidney injury,” AND “Cardiac surgery.” Additionally, reference lists of identified articles were manually screened to ensure inclusivity. No language restrictions were imposed. The complete search strategy is detailed in the Supplementary Digital Content.

### 2.2 Study selection

#### 2.2.1 Bibliometric analysis

Two independent investigators conducted the literature search and screening, resolving discrepancies through consensus. From the Web of Science database, 104 studies were initially identified for bibliometric analysis. Following rigorous evaluation, a final agreement rate of 90% was achieved, indicating strong inter-rater reliability (18). R Studio and VOS viewer software were employed to analyze geographical distribution (country/region contributions), cluster analysis (thematic groupings), and thematic mapping (emerging trends and research gaps). The analysis encompassed diverse study types, including basic research, clinical trials (RCT, cohort studies, case–control studies), reviews (both narrative and systematic), and meta-analyses.

#### 2.2.2 Meta-analysis of included RCTs

Furthermore, the studies retrieved from four databases were uploaded to reference management software, EndNote X9, where duplicate citations were removed. For the meta-analysis, the inclusion criteria for the current review were as follows: (1) the articles had to be published in English and be full-length articles; (2) case reports, protocols, letters, reviews and meta-analyses, conference abstracts, ongoing study and observational studies were excluded; (3) only RCTs with complete data were included. (4) The intervention arm had to investigate the use of DEX on AKI in cardiac surgery; (5) the control arm had to involve a placebo, or normal saline; and (6) the outcomes had to include the renal function outcome, or the incidence of AKI. The primary outcome was defined as the incidence of AKI according to different classification and creatinine clearance rates after surgery. The secondary outcomes included: patient demographics (age, comorbidities: diabetes mellitus, hypertension); intraoperative parameters (surgery duration, aortic cross-clamp time, CPB time); postoperative outcomes (ICU stay, mechanical ventilation duration, hospital stay, complications: bradycardia, hypotension, mortality).

### 2.3 Data extraction

Two reviewers independently extracted data using Microsoft Excel 2021, resolving discrepancies through discussion. Extracted variables included: study characteristics (publication year, first author, sample size), patient demographics (mean age, comorbidities), intervention details (DEX dosage, administration timing, surgery type), outcomes measures (AKI definition, follow-up duration). Categorical variables were reported as incidence rates, while continuous variables were expressed as mean (SD) or median (IQR).

### 2.4 Risk of bias and quality assessment

The Cochrane Risk of Bias Tool (RoB 2) was applied to evaluate bias across five domains: randomization process, deviations from intended interventions, missing outcome data, measurement of the outcome, and selection of the reported result (19). Each domain contains a series of signaling questions. Two reviewers independently assessed each study, categorizing bias as low, some concerns, or high. The Grading of Recommendations Assessment, Development, and Evaluation (GRADE) framework was used to rate evidence quality (low, moderate, or high) for each outcome (20).

### 2.5 Statistical analysis

The primary outcome was the incidence of AKI, analyzed as risk ratios (RR) with 95% confidence intervals (CI) using a random-effects model (Mantel–Haenszel statistical method) (21). For this continuous data, we calculated the mean difference (MD) with the corresponding 95% CI using a random-effects model. Results initially presented as medians and interquartile ranges (IQR) were transformed to means and SDs using the formula described by Hozo et al. (22). Assessed via *I*^2^ statistic (threshold: >50% = random-effects; ≤50% = fixed-effects) (23). Subgroup analyses were conducted to explore heterogeneity (e.g., DEX dosing regimens and different AKI definition) (24). Funnel plots and Egger test were constructed to assess potential publication bias and small-study effects for the primary outcome. For multi-arm trials, to avoid overestimation of sample size, participants were proportionally allocated: if one intervention group was compared to two control groups, the intervention group sample size was split proportionally to enable valid comparisons with each control group. Sensitivity analyses were performed using a leave-one-out approach to identify potential sources of heterogeneity influencing the primary outcome. Statistical significance was set at *p* < 0.05. Analyses were performed using Review Manager 5.4, R Studio, Vosviewer and GRADE Profiler 3.6.

## 3 Results

**Figure 1:**
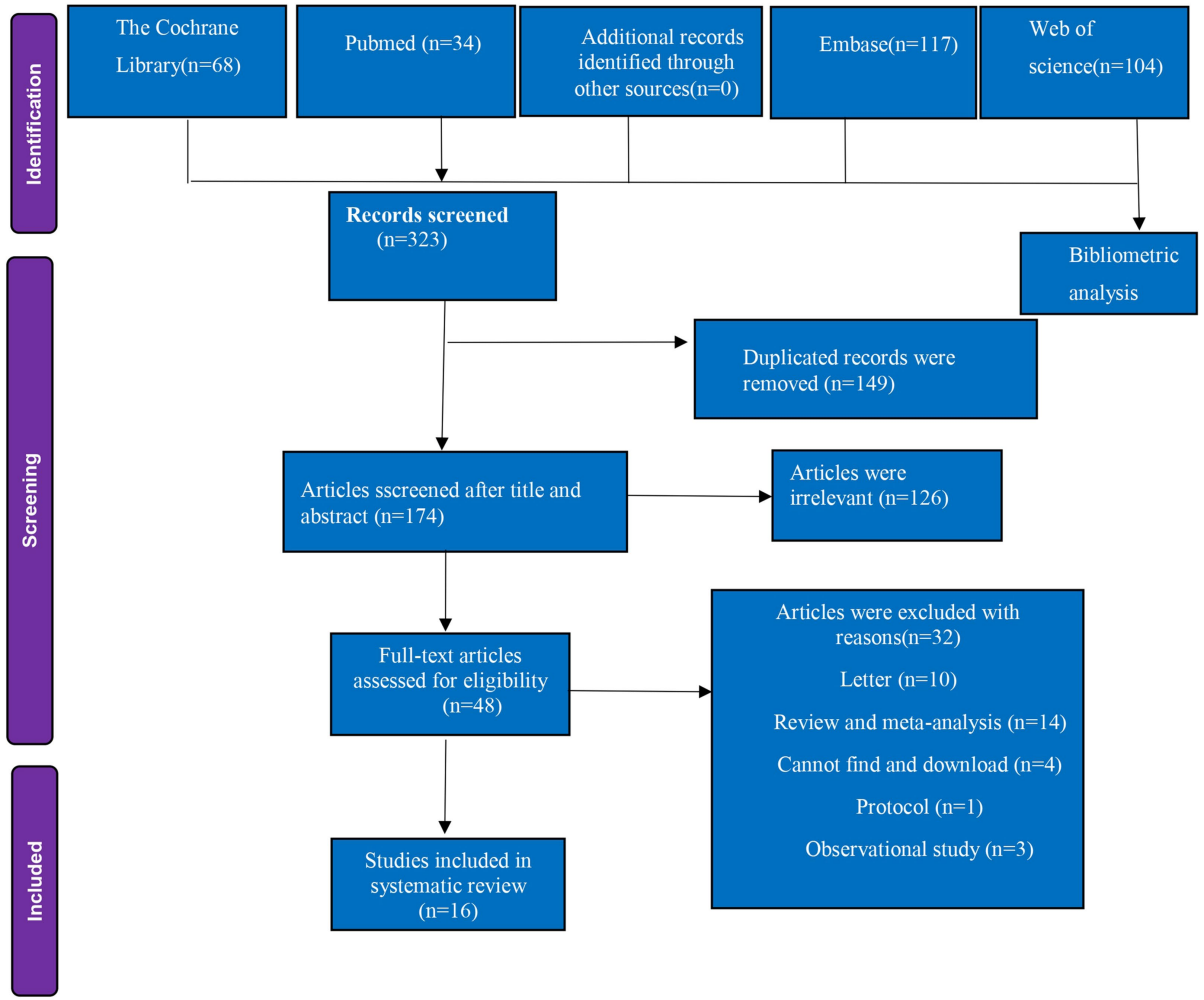
PRISMA flow diagram of included and excluded studies. PRISMA, Preferred Reporting Items for Systematic Reviews and Meta-Analyses.

The PRISMA flowchart (Figure 1) outlines the screening process. Initial searches yielded 323 citations, with 104 studies from Web of Science undergoing bibliometric analysis. Following a thorough review of full articles and subsequent exclusions, 16 RCTs involving 2,882 patients were identified as meeting the inclusion criteria as shown in the graphical abstract.

### 3.1 Bibliometric results

**Figure 2:**
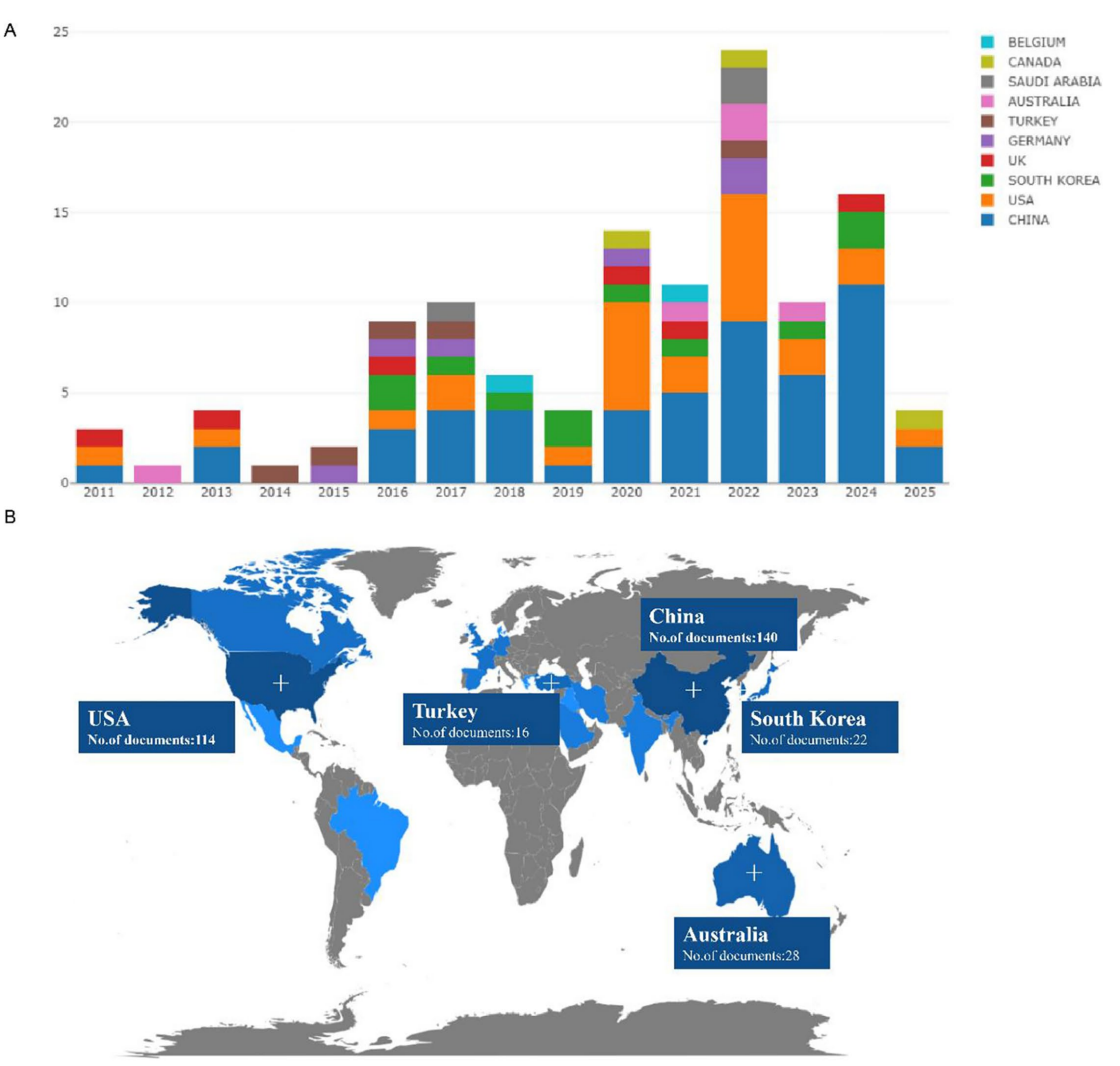
Publication trends and leading contributors on DEX and AKI in cardiac surgery (from Web of Science database). **(A)** The publication trends and timeline for studies investigating the effects of DEX on AKI in cardiac surgery patients; **(B)** The leading contributors of DEX on AKI in cardiac surgery patients. DEX, dexmedetomidine; AKI, acute kidney injury.

**Figure 3:**
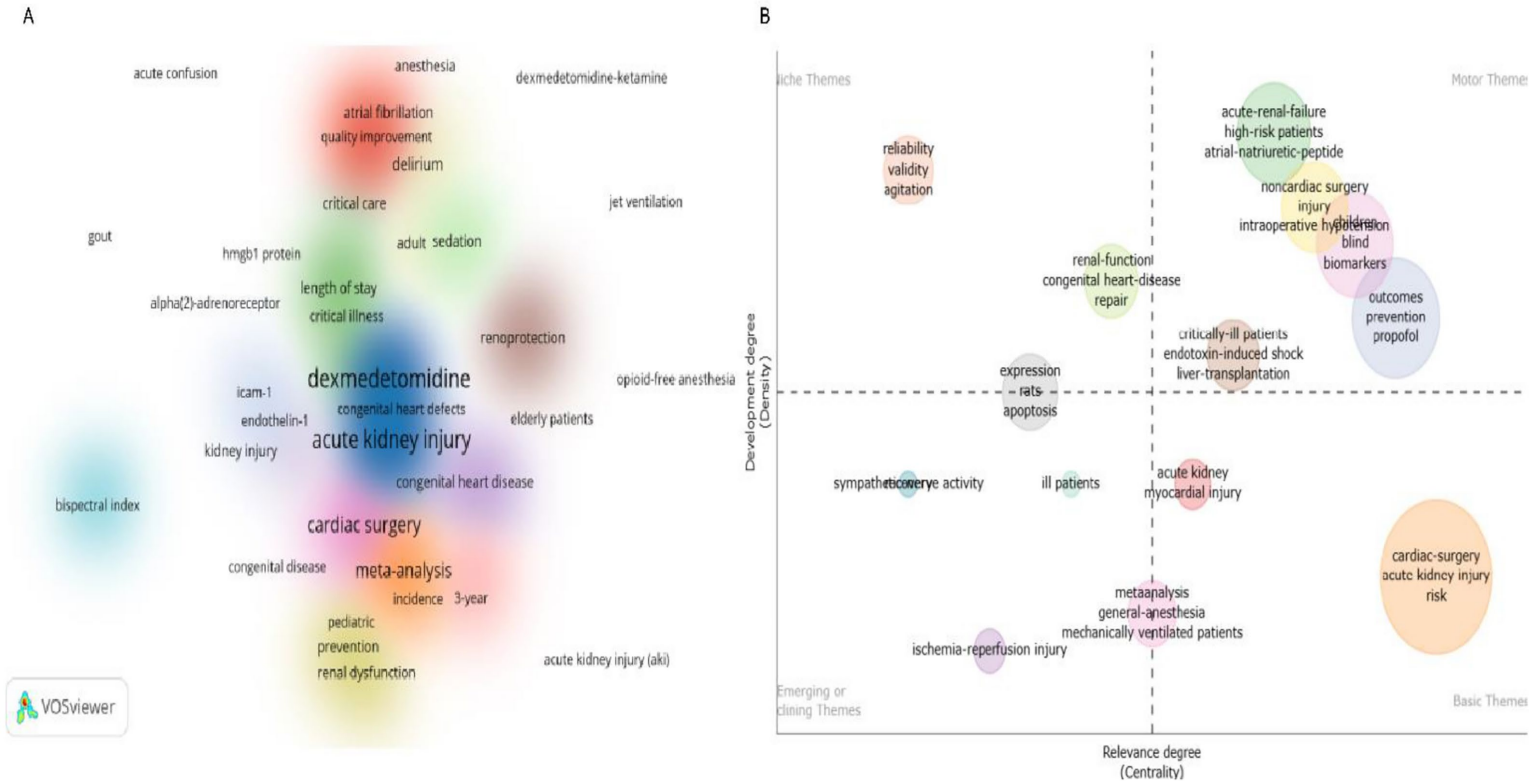
Research trends in DEX on AKI in patients undergoing cardiac surgery (from Web of Science database). **(A)** Analysis of research keywords in DEX and AKI in cardiac surgery; **(B)** Thematic evolution of DEX research: from organ protection to precision biomarkers in critical care. DEX, dexmedetomidine; AKI: acute kidney injury.

Studies exploring DEX-mediated renal protection against AKI in cardiac surgery patients were published from 2011 to 2025, with a notable surge in 2022 (Figure 2A). China and the U.S. lead this research domain, while Australia, South Korea, and the U.K. also contribute substantially (Figure 2B). Coupling clustering analysis clarified interstudy connections and developmental trajectories. Keyword analysis (Figure 3A) identified three core clusters: dominant themes (DEX applications, AKI/organ protection, clinical contexts), emerging trends/gaps (pediatric focus, delirium/neurological effects, techniques/comorbidities), and future research priorities (mechanistic studies, clinical trials, multidisciplinary approaches). A thematic map (Figure 3B) categorized related topics into four groups. Collectively, these visualizations comprehensively delineate the research landscape, highlighting key focus areas, academic influence, and emerging directions for future investigations.

### 3.2 Meta-analysis of included RCTs

Table 1 presents the characteristics and details of these RCTs (13, 14, 25–38). All 16 included articles were published between 2011 and 2024 and were authored in English. The geographical distribution of these studies is as follows: two from Turkey (25, 37), two from Republic of Korea (14, 26), five from China (13, 27–30), two from Egypt (31, 34), and one each from Finland (32), Russia (33), USA (35), Pakistan (36), and Iran (38) respectively. Twenty studies were designed with two arms (13, 14, 26–38), while one study featured a three-arm design (25). This diverse geographical representation highlights the global interest and contribution to research on this topic (see Table 2).

**Table 1:** Demographic information of included studies.

Study	Country	Registration numbers	No. of patients	Mean age	Male/female	Hypertension	Diabetes mellitus
Balkanay et al. (25)	Turkey	–	28 vs. 31 vs. 29	60.5 ± 8.6	65/23	74	39
Cho et al. (14)	Republic of Korea	–	100 vs. 100	64 ± 12 vs. 62 ± 13	45/55 vs. 51/49	50 vs. 41	22 vs. 17
Ham et al. (26)	Republic of Korea	ClinicalTrials.gov (NCT02698930)	32 vs. 31	56 (21–82) vs. 54 (23–79)	18/14 vs. 25/6	14 vs. 10	8 vs. 6
Qiu et al. (27)	China	ChiCTR2000041099	35 vs. 35	54.74 ± 11.15 vs. 55.74 ± 9.43	18/17 vs. 15/20	30 vs. 24	34 vs. 31
Tang et al. (28)	China	ChiCTR-IPR-14005656.	38 vs. 37	54 ± 7.7 vs. 56 ± 8.5	22/15 vs. 24/14	–	–
Wang et al. (13)	China	–	326 vs. 326	54.0 ± 11.8 vs. 53.8 ± 12.6	159/167 vs. 166/160	93 vs. 100	17 vs. 18
Zhai et al. (29)	China	ChiCTR-TRC-14004832	36 vs. 36	45 ± 10 vs. 47 ± 11	17/19 vs. 16/20	–	–
Zhang et al. (30)	China	ChiCTR2100051804	115 vs. 114	64.08 ± 6.96 vs. 65.11 ± 6.57	79/36 vs. 72/42	36 vs. 43	28 vs. 33
Ammar et al. (31)	Egypt	PACTR201507000984471	25 vs. 25	55.4 ± 7.1 vs. 59.1 ± 6.2	20/5 vs. 18/7	22 vs. 19	18 vs. 16
Leino et al. (32)	Finland	–	35 vs. 31	59.5 ± 8.5 vs. 62.4 ± 7.0	31/4 vs. 28/3	–	–
Likhvantsev et al. (33)	Russia	ClinicalTrials.gov (NCT03091166)	84 vs. 85	126 [101–168] vs. 117 [102–157]	62/22 vs. 60/25	64 vs. 70	38 vs. 36
Soliman et al. (34)	Egypt	–	75 vs. 75	58.37 ± 7.32 vs. 57.82 ± 7.65	37/40 vs. 38/35	38 vs. 35	21 vs. 25
Turan et al. (35)	USA	NCT02004613	398 vs. 396	63 ± 11 vs. 62 ± 12	266/130 vs. 287/107	264 vs. 269	91 vs. 74
Chohan et al. (36)	Pakistan	–	30 vs. 30	55.4 ± 9.2 vs. 55.2 ± 9.7	22/8 vs. 20/10	4 vs. 2	6 vs. 6
Göksedef et al. (37)	Turkey	–	49 vs. 37	58.0 ± 12.5 vs. 63.0 ± 10.2	35/14 vs. 28/9	36 vs. 27	14 vs. 15
Jannati et al. (38)	Iran	IRCT20141009019470N83	28 vs. 30	60.82 ± 6.47 vs. 57.7 ± 6.57	12/16 vs. 17/13	–	–

**Table 2:** Summary of the included studies.

Study	Surgery	Dexmedetomidine dose	Control arm	Time and duration of intervention or control	Primary outcomes	AKI definition	Follow up	Conclusions
Balkanay et al. (25)	CABG	1.4 μg/cc concentration	Placebo	1. At a speed of 0.04 μg/kg/h in the ICU follow-up period.	Serum NGAL levels	RIFLE	In hospital	DEX infusion for sedation after CABG under cardiopulmonary bypass can be useful in the prevention of kidney injury.
		2.8 μg/cc		2. Increased up to 0.5 μg/kg/h according to the needs of sedation continued for a maximum of 24 h.				
Cho et al. (14)	Valvular heart surgery	0.4 μg/kg/h	Equal placebo	Starting immediately after anesthetic induction and continuing for 24 h after surgery	Incidence of AKI during the first 48 postoperative	Based on acute kidney injury network criteria	In hospital	Perioperative infusion of DEX effectively reduced both the incidence and severity of AKI, and improved without untoward hemodynamic side effects.
Ham et al. (26)	Cardiac surgery	0.4 μg/kg/h	Equal normal saline	Infusion for 24 h after induction of anesthesia	The occurrence of AKI within 7 days post operation	1. 1.5 times the baseline within 7 days or a ≥ 0.3 mg dL^−^1 increase within 48 h in serum creatinine; 2. Initiation of RRT; 3. Urine output <0.5 mL kg^−^1 h^−^1 for over 6 h.	Until discharge or up to 30 days postoperatively	DEX administration for 24 h starting from induction of anesthesia significantly reduced the incidence of postoperative AKI without hemodynamic side effects.
Qiu et al. (27)	Cardiac valve surgery	0.6 μg/kg/h	Equal normal saline	Infusion from 10 min before anesthesia induction to 6 h after surgery	The incidence of AKI	KDIGO	In hospital	DEX may be considered as a way to reduce the incidence and severity of postoperative AKI.
Tang et al. (28)	Heart valve replacement surgery	loading doses of 1.0 μg/kg + continuous infusion of 0.166 mL/kg/h (0.3 μg/kg/h) for maintenance	Equal normal saline	Pretreatment	The incidence of AKI	KDIGO		These results suggested that DEX pretreatment attenuates MI/R injury-induced AKI by relieving the ER stress.
Wang et al. (13)	Heart valve surgery	0.6 μg/kg over 10 min and continuous infusion 0.4 μg/kg/h until the end of surgery	Equal normal saline	Until the end of surgery.	The incidence of POD	–	In hospital	Intra-operative DEX infusion did not reduce the incidence of delirium after cardiac valve surgery but might impair renal function.
Zhai et al. (29)	Cardiac valve replacement	0.6 μg/kg was administered in at 15 min before anesthesia induction, followed by of 0.2 μg/kg/h	Equal normal saline	Until the end of operation.		RIFLE	In hospital	DEX may attenuate the renal injury and decrease the incidence of AKI.
Zhang et al. (30)	CABG	0.5 μg/kg over 10 min, then 0.4 μg/kg/h until the end of surgery	Equal normal saline	Until the end of surgery	Incidence of AKI within 96 h after surgery	–		DEX reduces AKI, potentially by regulating metabolic disorders and reducing oxidative stress.
Ammar et al. (31)	Elective cardiac surgeries	Continuous initiated 5 min before cardiopulmonary bypass (1 μg/kg over 15 min, followed by 0.5 μg/kg/h)	Equal normal saline	Until 6 h after surgery	Myocardial‑specific proteins and urinary‑specific kidney proteins	–	In hospital and postpositive 30 days	DEX reduced cardiac and renal injury as evidenced by lower concentration of myocardial-specific proteins, kidney-specific urinary proteins, and pro-inflammatory cytokines.
Leino et al. (32)	CABG	0.60 ng/mL	Equivolume infusion of placebo	After anesthetic induction and last for 4 h	Creatinine clearance at 12–24 h prior to surgery, and 0–24 h and 24–48 after urinary catheter insertion after induction of anesthesia	RIFLE	In hospital	DEX did not alter renal function but was associated with an increase in urinary output.
Likhvantsev et al. (33)	Elective cardiac surgery	Started in the operating room (0.7 μg /kg/h) and continued into the ICU (0.4 μg /kg/h)	Equivolume infusion of placebo	Started in the operating room and continued into the ICU	The incidence of POD	KDIGO	In hospital and postpositive 30 days	DEX administered during reduced the incidence of POD and decreased the length of stay in the intensive care unit and hospital.
Soliman et al. (34)	Egypt	Loading dose of 1 μg/kg over 15 min before induction and maintained as an infusion of 0.3 μg/kg/h to the end of the procedure	Equal volume of normal saline	Started before induction and to the end of the procedure	–	–	In hospital	DEX is safe and effective in patients undergoing aortic vascular surgery.
Turan et al. (35)	USA	At a rate of 0.1 μg/kg/h increased to 0.2 μg/kg/h at the end of bypass, postoperatively increased to 0.4 μg/kg/h was maintained until 24 h	Equal volume of normal saline	Started before the surgical incision and maintained until 24 h	Atrial fibrillation and delirium occurring between ICU admission and the earlier of postoperative day 5 or hospital discharge	–	In hospital	DEX infusion, initiated at anaesthetic induction and continued for 24 h, did not decrease postoperative atrial arrhythmias or delirium in patients recovering from cardiac surgery.
Chohan et al. (36)	Pakistan	0.4 μg/kg/h from induction of anesthesia for 24 h	Equal volume of normal saline	Started from induction of anesthesia for 24 h	Perioperative serum creatinine (mg/dL)	KDIGO	In hospital	DEX infusion significantly reduced incidence of AKI.
Göksedef et al. (37)	Turkey	0.04–0.5 μg/kg/h		Administered maintained at postoperatively in the ICU and increased so that in a maximum 24 h period				Low dose DEX has no major effect on urine output and renal indices such as urea, creatinine and creatinine clearances. However, it may have a positive effect on renal functions when total dose is uptitrated, particularly.
Jannati et al. (38)	CABG	0.5 μg/kg/h	Equal volume of normal saline	Started after general anesthesia induction and until the end of the operation	–	–	In hospital	DEX does not affect the renal function of patients undergoing CABG.

CABG, coronary artery bypass grafting; AKI, acute kidney injury; DEX, dexmedetomidine; ICU, intensive care unit; NGAL, neutrophil gelatinase-associated lipocalin; RIFLE, risk, injury, failure, loss, and end-stage; RRT, renal replacement therapy; MI/R, myocardial ischemia reperfusion; ER, endoplasmic reticulum; POD, postoperative delirium; KDIGO, kidney disease: improving global outcomes.

#### 3.2.1 Renal protection of DEX after cardiac surgery

The incidence of AKI was defined according to the RIFLE, Acute Kidney Injury Network criteria, or KDIGO postoperatively. A total of 11 studies demonstrated the incidence of AKI of DEX in patients undergoing cardiac surgery, compared with placebo or normal saline (13, 14, 26–30, 33–36). DEX significantly reduced AKI risk [RR 0.58; 95% CI 0.37 to 0.91; *I*^2^ = 74%; *p* = 0.002] (Supplementary Figure 1). Considering the *I*^2^ > 50%, a random model was applied. Subgroup analyses based on 0.4 μg/kg/h were maintained of DEX vs. placebo or normal saline revealed the following findings: there is no significant reduction in AKI incidence [RR 0.65; 95% CI 0.36 to 1.17; *I*^2^ = 85%; *p* = 0.15] (13, 14, 25, 26, 30, 33, 35, 36) (Supplementary Figure 2). However, 0.6–1.0 μg/kg/h of DEX vs. placebo or normal saline revealed a significant difference favor of DEX [RR 0.43; 95% CI 0.26 to 0.71; *I*^2^ = 0%; *p* = 0.001] (27–29, 34) (Supplementary Figure 2). Consistent renoprotective effects on the KDIGO definitions [RR 0.39; 95% CI 0.24 to 0.66; *I*^2^ = 14%; *p* = 0.0004] (27, 28, 33, 36) (Supplementary Figure 3). Moreover, the urine output at postoperative 24 h is favored of DEX compared with control [MD 159.7; 95% CI 106.12 to 213.29; *I*^2^ = 34%; *p* < 0.00001] (14, 25, 28, 30, 36, 37), but no significant difference at 48 h [MD −14.55; 95% CI −165.53 to 136.42; *I*^2^ = 94%; *p* = 0.85] (14, 28, 36) (Supplementary Figure 4).

#### 3.2.2 The average age, preoperative DM, and HTN

There were no significant differences between the DEX group and the comparator groups in terms of age [MD −0.12; 95% CI −0.83 to 0.59; *I*^2^ = 30%; *p* = 0.75] (13, 14, 26–38) (Supplementary Figure 5), DM [RR 1.06; 95% CI 0.93 to 1.20; *I*^2^ = 0%; *p* = 0.42] (13, 14, 26, 27, 30, 31, 33–37), or HTN [RR 1.00; 95% CI 0.93 to 1.07; *I*^2^ = 0%; *p* = 0.96] (13, 14, 26, 27, 30, 31, 33–37) (Supplementary Figure 6).

#### 3.2.3 The duration of surgery, aortic cross-clamp, and CPB time

No significant differences were observed between the DEX group and the comparator groups in the duration of surgery (minutes) [MD −1.17; 95% CI −9.42 to 6.01; *I*^2^ = 82%; *p* = 0.66] (13, 14, 26, 27, 29–32, 34, 36), aortic cross-clamp (minutes) [MD −2.52; 95% CI −5.59 to 0.55; *I*^2^ = 81%; *p* = 0.11] (13, 14, 25–31, 33, 34, 36, 38), or CPB time (minutes) [MD −2.60; 95% CI −7.66 to 2.45; *I*^2^ = 88%; *p* = 0.31] (13, 14, 26–31, 33, 34, 36, 38)(Supplementary Figure 7).

#### 3.2.4 The duration of ICU, mechanical ventilation, and hospital stay

Significant differences were observed between the DEX group and the comparator groups in the duration of ICU (h) [MD −1.23; 95% CI −2.17 to −0.30; *I*^2^ = 93%; *p* = 0.01] (13, 14, 25–28, 30, 31, 33, 35–37), mechanical ventilation (h) [MD −1.24; 95% CI −2.15 to −0.33; *I*^2^ = 97%; *p* = 0.008] (14, 25–33, 37, 38), and the hospital stay (day) [MD −0.33; 95% CI −0.54 to −0.13; *I*^2^ = 86%; *p* = 0.001] (14, 25–28, 30, 31, 33, 35, 37) (Supplementary Figure 8).

#### 3.2.5 Postoperative complications

No significant difference were observed between the DEX group and the comparator groups in the postoperative bradycardia [RR 0.97; 95% CI 0.58 to 1.16; *I*^2^ = 21%; *p* = 0.89] (25, 27, 30, 34–36), hypotension [RR 1.10; 95% CI 0.79 to 1.53; *I*^2^ = 70%; *p* = 0.58] (13, 25, 27, 34–36), in-hospital mortality [RR 0.35; 95% CI 0.09 to 1.35; *I*^2^ = 0%; *p* = 0.13] (14, 26, 34, 35), and postoperative 30-day mortality [RR 1.10; 95% CI 0.15 to 7.02; *p* = 0.99] (31, 33) (Supplementary Figure 9).

#### 3.2.6 Publications bias

According to this meta-analysis, the results showed high heterogeneity (*I*^2^ > 50). A sensitivity analysis was performed via the leave-one-out approach, revealing no significant changes in pooled effect size in the primary outcome of the incidence of AKI (Supplementary Table 1). The funnel plot revealed a small asymmetry in the incidence of AKI (Supplementary Figure 10). Moreover, Egger’s test performed in the incidence of AKI (Intercept = 0.2246, *p* = 0.07, RR = 0.788), revealed no significant publication bias, and a low risk of publication bias for the study result. Using the GRADE methodology, we assessed evidence for the primary and secondary outcomes: The quality of evidence for the main outcomes was moderate to high (Supplementary Table 2). Publication bias was evaluated utilizing the Cochrane Risk of Bias Tools (RoB2). Of included studies, nine (56.25%) were categorized as having low risk of bias, two (12.5%) were deemed to have unclear risk of bias, and five (31.25%) were classified as having high risk of bias (Supplementary Table 3).

## 4 Discussion

Current study demonstrated China, and the USA dominated research output, reflecting regional disparities in funding and clinical priorities. Emerging themes included pediatric applications and biomarker-guided therapy (e.g., NGAL, cystatin C), which remain underexplored in RCTs. Furthermore, the meta-analysis of 16 RCTs (*n* = 2,882) demonstrates that DEX significantly reduces the incidence of AKI in cardiac surgery patients, with notable improvements in postoperative recovery, including shorter ICU stays, mechanical ventilation duration, and hospital stays. However, its effects on mortality and intraoperative parameters remain inconclusive. Moreover, we contextualize these findings, explore mechanistic insights, address limitations, and propose future research directions.

Current results align with prior meta-analyses reporting DEX’s renoprotective effects (39–41), but contrast with studies showing no benefit (13, 35). This discrepancy may stem from dosing heterogeneity. Subgroup analysis demonstrated that DEX regimens other than 0.4 μg/kg/h exerted a significant renoprotective effect (RR 0.43; *p* = 0.001), whereas the 0.4 μg/kg/h dose failed to yield a statistically significant benefit (RR 0.65; *p* = 0.15). These findings imply that the 0.4 μg/kg/h DEX dosage may be subtherapeutic with respect to renoprotection; however, the limited sample size of the moderate-dose subgroup and high between-study heterogeneity necessitate prudent interpretation of these results (15). Although the included RCTs employed heterogeneous methods and tools to define AKI, which may have confounded the findings of the present study, the 2022 AKI Consensus Definition has highlighted that current approaches to AKI definition using routinely collected clinical data remain inconsistent and inadequately characterized in the existing literature. Consensus among experts has not been reached regarding multiple aspects of AKI definition and the description of its sequelae. Therefore, the KDIGO guidelines should be extended to include a standardized definition for how AKI should be defined when using routinely collected data (42). Furthermore, subgroup analyses stratified by AKI diagnostic criteria demonstrated a consistent renoprotective effect when the KDIGO definition was applied (RR 0.39; *p* = 0.0004). DEX shortened ICU stay (*p* = 0.01), mechanical ventilation duration (*p* = 0.008), and hospital stays (*p* = 0.01), consistent with findings from Li et al. (15). These benefits likely reflect DEX’s sedative-sparing effects and reduced delirium incidence (40, 43). However, the lack of mortality reduction underscores the need for larger trials powered by hard endpoints.

Tang et al. (28) combined clinical and preclinical study revealed that DEX pretreatment attenuated AKI and oxidative stress as well as postischemic myocardial injury in patients. Accordingly, animal results suggested DEX reduced cellular injury and improved postischemic myocardial and renal function. Moreover, previous study also indicated DEX use was associated with reduced serum levels of NSE, S-100β within 24 h of the surgery. Also, DEX use was associated with reduced levels of interleukin-6 (44). The application of DEX in cardiac surgery with CPB can reduce interleukin-6, tumor necrosis factor-α levels to a certain extent and shorten the length of ICU stay (45). There is no significant in postoperative bradycardia, hypotension, and mortality rate in DEX use. However, a previous study revealed is need to be caution the use DEX associated bradycardia and hypotension (43, 45).

### 4.1 Limitations of current study

This study has several limitations needed to be addressed. First, the heterogeneity in AKI definitions, included studies used varying criteria (RIFLE, AKIN, KDIGO) (14, 25, 27–29, 32, 33, 36), complicating cross-trial comparisons (42). For example, KDIGO’s inclusion of urinary output may overestimate AKI incidence compared to RIFLE. Second, DEX regimens differed in loading doses (0.5–1.0 μg/kg/h), maintenance durations (6–24 h), and timing (pre- vs. intraoperative). Such variability confounds dose–response conclusions (15). Third, of all 16 RCTs enrolled patients undergoing cardiac surgery with CPB, the use of DEX on on-pump or off-pump cardiac surgery may pose different effect on renoprotection. Last but not the least, while most RCTs had low bias in randomization, deviations in blinding (e.g., unblinded clinicians) raised “some concerns” more than 30% of studies. Funnel plot asymmetry suggests underreporting of negative results, potentially inflating effect sizes.

## 5 Conclusion

The bibliometric insights highlight the dominance of Chinese and American research, with emerging focus on pediatric and mechanistic studies. Future research should standardize dosing protocols and incorporate biomarker-driven approaches to personalize therapy. Moreover, the meta-analysis confirms that DEX significantly reduces AKI incidence and enhances postoperative recovery in cardiac surgery patients, particularly at doses of 0.6–1.0 μg/kg/h significantly reduces AKI incidence, while the effectiveness of 0.4 μg/kg/h remains inconclusive due to limited sample size and high heterogeneity in this subgroup. Clinically, these findings advocate DEX’s inclusion in perioperative guidelines but caution against uniform dosing.

## Data Availability

The original contributions presented in the study are included in the article/Supplementary material, further inquiries can be directed to the corresponding author.
