# Supplementary material for: Perioperative dexmedetomidine and renal outcomes in adult cardiac surgery: an updated systematic review and meta-analysis
Source: Front Med (Lausanne). 2026 Jan 16;12:1737121. doi: 10.3389/fmed.2025.1737121 (PMC12855419; doi:10.3389/fmed.2025.1737121)
Supplement: Supplementary file 1 [file Table_1.doc]

Supplmentary-Table1. The sensitivity analysis of the incidence of acute kidney injury between DEX and control in patients undergoing cardiac surgery.

| Study | Z effect | RR | 95%CI upper | 95%CI lower | I^2^ | P value |
| --- | --- | --- | --- | --- | --- | --- |
| Cho., et al. 2016 | 2.04 | 0.61 | 0.38 | 0.98 | 73% | 0.04* |
| Ham., et al. 2024 | 2.00 | 0.63 | 0.40 | 0.99 | 74% | 0.05* |
| Qiu., et al. 2023 | 2.06 | 0.62 | 0.39 | 0.98 | 75% | 0.04* |
| Tang., et al. 2020 | 2.07 | 0.62 | 0.40 | 0.98 | 75% | 0.04* |
| Wang., et al 2023 | 3.14 | 0.52 | 0.34 | 0.78 | 59% | 0.002* |
| Zhai., et al. 2017 | 2.10 | 0.61 | 0.38 | 0.97 | 76% | 0.04* |
| Zhang., et al. 2024 | 2.11 | 0.58 | 0.35 | 0.96 | 75% | 0.03* |
| Likhvantsev., et al 2021 | 2.41 | 0.56 | 0.35 | 0.90 | 77% | 0.02* |
| Soliman., et al. 2016 | 2.29 | 0.58 | 0.36 | 0.92 | 77% | 0.02* |
| Turan., et al. 2020 | 2.69 | 0.52 | 0.32 | 0.84 | 72% | 0.007* |
| Chohan., et al.2022 | 2.00 | 0.63 | 0.40 | 0.99 | 74% | 0.05* |

DEX: Dexmedetomidine.

Supplementary Table 2. Quality of evidence assessment for main results with GRADE.

| Outcomes | DEX *vs.* control | Relative RR or MD with 95% CI | Quality of the evidence |
| --- | --- | --- | --- |
| Primary outcome | | | |
| Incidence of AKI | 160/1269 (12.6%) *vs.*204/1265 (16.1%) | 0.58 (0.37, 0.91) | ⨁⨁⨁⨁ High |
| Secondary outcomes | | | |
| Postoperative 24 h urine output (ml/24 h) | 361 *vs.*377 | 159.7 (106.12, 213.29) | ⨁⨁⨁◯ Moderate |
| Postoperative 48 h urine output (ml/24 h) | 168 *vs.*167 | -14.55 (-165.33,136.42) | ⨁⨁⨁◯ Moderate |
| Mean age | 1406 *vs.*1388 | -0.12 (-0.83,0.59) | ⨁⨁⨁⨁ High |
| Diabetes mellitus | 297/1269 (23.4%) *vs.*277/1254 (22.1%) | 1.06 (0.93,1.20) | ⨁⨁⨁⨁ High |
| Hypertension | 653/1269(51.5%) *vs.*644/1254 (51.4%) | 1.00 (0.93,1.07) | ⨁⨁⨁⨁ High |
| Surgery time | 809 *vs.*803 | -1.71 (-9.42,6.01) | ⨁⨁⨁⨁ High |
| Aortic cross-clamp time | 953 *vs.*983 | -2.52 (-5.59,0.55) | ⨁⨁⨁⨁ High |
| CPB time | 849 *vs.*849 | -2.6 (-7.66,2.45) | ⨁⨁⨁⨁ High |
| ICU stays | 1261 *vs.*1275 | -1.23 (-2.17, -0.30) | ⨁⨁⨁⨁ High |
| Mechanical ventilation time | 832 *vs.*846 | -1.24 (-2.15, -0.33) | ⨁⨁⨁⨁ High |
| Hospital stays | 1131 *vs.*1144 | -0.33 (-0.54, -0.13) | ⨁⨁⨁⨁ High |
| Postoperative bradycardia | 59/682(8.7%) *vs.*66/709(9.3%) | 0.97 (0.58,1.61) | ⨁⨁⨁⨁ High |
| Postoperative hypotension | 307/893(34.4%) *vs.*247/921(26.8%) | 1.10 (0.79,1.53) | ⨁⨁⨁⨁ High |
| Postoperative in-hospital mortality | 2/605(0.3%) *vs.*8/602(1.3%) | 0.35 (0.09,1.35) | ⨁⨁⨁⨁ High |
| Postoperative 30-day mortality | 2/109(1.8%) *vs.*2/110(1.8%) | 1.01 (0.15,7.02) | ⨁⨁⨁⨁ High |

GRADE: Grading of Recommendations Assessment, Development, and Evaluation; AKI: Acute kidney injury; MD: Mean difference; RR: Relative risk; CI: confidence intervals; CPB: cardiopulmonary bypass; ICU: Intensive care unit.

Supplmentary-Table3. Risk bias assessment of included RCTs.

| Study | Bias arising from the randomization process | Bias due to deviations from intended interventions | Bias due to missing outcome data | Bias in measurement of the outcome | Bias in selection of the reported result | Overall bias |
| --- | --- | --- | --- | --- | --- | --- |
| Balkanay., et al. 2015 | Y | Y | N | PY | PY | High risk of bias |
| Cho., et al. 2016 | Y | Y | N | PY | PY | High risk of bias |
| Ham., et al. 2024 | N | N | N | N | N | Low risk of bias |
| Qiu., et al. 2023 | N | N | N | N | N | Low risk of bias |
| Tang., et al. 2020 | Y | Y | N | N | PY | Unclear risk of bias |
| Wang., et al 2023 | N | N | N | N | N | Low risk of bias |
| Zhai., et al. 2017 | N | N | N | PY | N | Low risk of bias |
| Zhang., et al. 2024 | N | N | N | N | N | Low risk of bias |
| Ammar., et al. 2016 | N | N | N | N | N | Low risk of bias |
| Leino., et al. 2011 | Y | Y | N | PY | PY | High risk of bias |
| Likhvantsev., et al 2021 | N | N | N | N | N | Low risk of bias |
| Soliman, 2016 | Y | Y | N | PY | PY | High risk of bias |
| Turan., et al. 2020 | N | N | N | N | N | Low risk of bias |
| Chohan., et al.2022 | N | N | N | N | N | Low risk of bias |
| Göksedef., et al.2013 | Y | Y | N | PY | PY | High risk of bias |
| Jannati., et al.2021 | N | N | N | PY | PY | Unclear risk of bias |

Y=yes; PY=probably yes; N=no.
