# Supplementary material for: Perioperative dexmedetomidine and renal outcomes in adult cardiac surgery: an updated systematic review and meta-analysis
Source: Front Med (Lausanne). 2026 Jan 16;12:1737121. doi: 10.3389/fmed.2025.1737121 (PMC12855419; doi:10.3389/fmed.2025.1737121)
Supplement: Supplementary file 2 [file Data_Sheet_1.doc]

Supplementary Figure 1. Forest plot of incidence of AKI between DEX and control groups. AKI: Acute kidney injury; DEX: Dexmedetomidine.

Supplementary Figure 2. Forest plot of incidence of AKI between DEX and control groups (different does of DEX). AKI: Acute kidney injury; DEX: Dexmedetomidine.

Supplementary Figure 3. Forest plot of incidence of AKI between DEX and control groups (different AKI definition). AKI: Acute kidney injury; DEX: Dexmedetomidine.

Supplementary Figure 4. Forest plot of postoperative urine output between DEX and control groups. DEX: Dexmedetomidine.

Supplementary Figure 5. Forest plot of mean age between DEX and control groups. DEX: Dexmedetomidine.

Supplementary Figure 6. Forest plot of preoperative comorbidities between DEX and control groups. DEX: Dexmedetomidine.

Supplementary Figure 7. Forest plot of the duration of surgery, aortic cross-clamp, and CPB time between DEX and control groups. DEX: Dexmedetomidine; CPB: Cardiopulmonary bypass.

Supplementary Figure 8. Forest plot of the duration of ICU, mechanical ventilation, and hospital stay between DEX and control groups. DEX: Dexmedetomidine; ICU: Intensive care unit.

Supplementary Figure 9. Forest plot of postoperative complications between DEX and control groups. DEX: Dexmedetomidine.

Supplementary Figure 10. Funnel plot of incidence of AKI between DEX and control groups. AKI: Acute kidney injury; DEX: Dexmedetomidine.
