# Supplementary material for: Perioperative dexmedetomidine and renal outcomes in adult cardiac surgery: an updated systematic review and meta-analysis
Source: Front Med (Lausanne). 2026 Jan 16;12:1737121. doi: 10.3389/fmed.2025.1737121 (PMC12855419; doi:10.3389/fmed.2025.1737121)
Supplement: Supplementary file 3 [file Data_Sheet_2.doc]

Pubmed,2025-04-26,34

| #1: **((Dexmedetomidine Hydrochloride [Title/Abstract]) OR (Hydrochloride, Dexmedetomidine [Title/Abstract])) OR ("Dexmedetomidine"[Mesh])** |
| --- |
| #2: **((((("Cardiac Surgical Procedures"[Mesh]) OR (Heart Surgical Procedure [Title/Abstract])) OR (Heart Surgical Procedures [Title/Abstract])) OR (Cardiac Surgical Procedure [Title/Abstract])) OR (Cardiac Surgery [Title/Abstract])) OR (Heart Surgery [Title/Abstract])** |
| #3: #1 and #2 |
| #4: **((((((((((("Acute Kidney Injury"[Mesh]) OR (Acute Kidney Injuries[Title/Abstract])) OR (Acute Renal Injury[Title/Abstract])) OR (Acute Renal Injuries[Title/Abstract])) OR (Acute Kidney Failures[Title/Abstract])) OR (Acute Kidney Failure[Title/Abstract])) OR (Acute Renal Failure[Title/Abstract])) OR (Acute Renal Failures[Title/Abstract])) OR (Acute Renal Insufficiencies[Title/Abstract])) OR (Acute Kidney Insufficiency[Title/Abstract])) OR (Acute Renal Insufficiency[Title/Abstract])) OR (Acute Kidney Insufficiencies[Title/Abstract])** |
| #5: #3 and #4 |

Web of science,2025-04-26,104

| #1: ((TS= (Dexmedetomidine Hydrochloride)) OR TS= (Hydrochloride, Dexmedetomidine)) OR TS=(Dexmedetomidine) |
| --- |
| #2: (((((TS= (Cardiac Surgical Procedures)) OR TS= (Heart Surgical Procedure)) OR TS= (Heart Surgical Procedures)) OR TS= (Cardiac Surgical Procedure)) OR TS= (Cardiac Surgery)) OR TS= (Heart Surgery) |
| #3: #1 and #2 |
| #4: **(((((((((((TS=(Acute Kidney Injury)) OR TS=(Acute Kidney Injuries)) OR TS=(Acute Renal Injury)) OR TS=(Acute Renal Injuries)) OR TS=(Acute Kidney Failures)) OR TS=(Acute Kidney Failure)) OR TS=(Acute Renal Failure)) OR TS=(Acute Renal Failures)) OR TS=(Acute Renal Insufficiencies)) OR TS=(Acute Kidney Insufficiency)) OR TS=(Acute Renal Insufficiency)) OR TS=(Acute Kidney Insufficiencies)** |
| #5: #3 and #4 |

Cochrane library,2025-04-26,68

| #1:(Dexmedetomidine):ti,ab,kw **OR** (Dexmedetomidine Hydrochloride):ti,ab,kw **OR** (Hydrochloride, Dexmedetomidine):ti,ab,kw |
| --- |
| #2:(Cardiac Surgical Procedures):ti,ab,kw **OR** (Heart Surgical Procedure):ti,ab,kw **OR** (Heart Surgical Procedures):ti,ab,kw **OR** (Cardiac Surgical Procedure):ti,ab,kw **OR** (Cardiac Surgery):ti,ab,kw **OR** (Heart Surgery):ti,ab,kw |
| #3: #1 and #2 |
| #4:(Acute Kidney Injury):ti,ab,kw **OR** (Acute Kidney Injuries):ti,ab,kw **OR** (Acute Renal Injuries):ti,ab,kw **OR** (Acute Kidney Failures):ti,ab,kw **OR** (Acute Kidney Failure):ti,ab,kw **OR** (Acute Renal Failure):ti,ab,kw |
| #5:#3 and #4 |

Embase,2025-04-26,117

| **#1:'dexmedetomidine'**/exp OR **dexmedetomidine'** OR **dexmedetomidine'** OR **'dexmedetomidine hydrochloride'** |
| --- |
| **#2:'heart surgery'** OR**' cardiac surgery '**OR **cardiac surgery associated acute kidney injury'** |
| #3:#1 and #2 |
| **#4:'acute kidney failure'** OR **'acute kidney injury'** |
| #5:#3 and #4 |
